# Assessment of the scope, completeness, and consistency of various drug information resources related to COVID-19 medications in pregnancy and lactation

**DOI:** 10.1186/s12884-023-05609-2

**Published:** 2023-04-27

**Authors:** Javedh Shareef, Sathvik Belagodu Sridhar, Mullaicharam Bhupathyraaj, Atiqulla Shariff, Sabin Thomas, Mohammed Salim Karattuthodi

**Affiliations:** 1 Department of Clinical Pharmacy & Pharmacology, RAK College of Pharmacy, RAK Medical & Health Sciences University, Ras Al Khaimah, United Arab Emirates; 2 College of Pharmacy, National University of Science and Technology, Muscat, 130 Oman; 3 Department of Pharmacy Practice, JSS College of Pharmacy, JSS Academy of Higher Education & Research, Mysuru, Karnataka India; 4 School of Pharmacy, College of Health Sciences, University of Nizwa, Nizwa, 616 Oman; 5 Department of Pharmacy Practice, Manipal College of Pharmaceutical Sciences, Manipal Academy of Higher Education, Manipal, Karnataka India

**Keywords:** Medication information, Fetal risk, Consistency, Treatment guidelines, Teratogenicity, Evidence-based information

## Abstract

**Background:**

Drug use in pregnancy and lactation is challenging. It becomes more challenging in pregnant and lactating women with certain critical clinical conditions such as COVID-19, because of inconsistent drug safety data. Therefore, we aimed to evaluate the various drug information resources for the scope, completeness, and consistency of the information related to COVID-19 medications in pregnancy and lactation.

**Methods:**

Data related to COVID-19 medications from various drug information resources such as text references, subscription databases, and free online tools were used for the comparison. The congregated data were analyzed for scope, completeness, and consistency.

**Results:**

Scope scores were highest for Portable Electronic Physician Information Database (PEPID), Up-to-date, and drugs.com compared to other resources. The overall completeness scores were higher for Micromedex and drugs.com (*p* < 0.05 compared to all other resources). The inter-reliability analysis for overall components by Fleiss kappa among all the resources was found to be 'slight' (*k* < 0.20, *p* < 0.0001). The information related to the older drugs in most of the resources, provides in-depth details on various components such as pregnancy safety, clinical data related to lactation, the effect of the drug distribution into breast milk, reproductive potential/infertility risk and the pregnancy category/recommendations. However, the information related to these components for newer drugs was superficial and incomplete, with insufficient data and inconclusive evidence, which is a statistically significant observation. The strength of observer agreement for the various COVID-19 medications ranged from poor to fair and moderate for the various recommendation categories studied.

**Conclusion:**

This study reports discrepancies in the information related to pregnancy, lactation, drug level, reproductive risk, and pregnancy recommendations among the resources directing to refer to more than one resource for information about the safe and quality use of medications in this special population.The present study also emphasizes the need for development of comprehensive, evidence-based, and precise information guide that can promote safe and effective drug use in this special population.

**Supplementary Information:**

The online version contains supplementary material available at 10.1186/s12884-023-05609-2.

## Background

Drug use during pregnancy and breastfeeding is often unavoidable and sometimes produces a severe risk to the woman, fetus, and newborn on exposure. This calls for a defined surveillance system, and caution should be exercised while prescribing drugs during pregnancy since some medications are teratogenic and have adverse fetal outcomes [1, 2]. The thalidomide tragedy has drawn the attention that the drugs cross the placental barrier and reach the fetus's systemic circulation causing potentially irreversible harmful effects [2, 3]. In addition, it is well known that physiologic and physical changes accompany pregnant women due to increased reproductive hormonal secretions and fetal growth [1].

These changes cause alteration in the drug action and pharmacokinetic profile of drugs resulting in increased volume of distribution, high degree of lipid solubility, decreased plasma concentration, reduced plasma half-life, and low level of protein binding. These factors enable the unbound drugs to cross the placental barrier harming the fetus [4, 5]. Some medications are indeed likely to be excreted in breast milk in varying amounts exerting a high risk for newborns and infants [6]. Specific properties of the medicines such as bioavailability, lipid solubility, molecular weight, protein binding, dose, frequency, duration of exposure, and amount of milk consumed are some factors that enhance the drug transfer into breast milk predisposing to neonatal toxicity [7].

Coronavirus disease (COVID-19) continues to spread at an accelerated rate globally and poses a significant threat to human health [8, 9]. The concerns were raised globally when the first case of COVID-19 was reported, leading to adverse outcomes and increased mortality rates in pregnant women [8, 10, 11]. A woman's physiological and immunological changes during pregnancy predispose her toward significant respiratory complications and susceptibility to specific intracellular pathogens [12, 13]. Evidence shows that pregnant women infected with COVID-19 have severe health impacts with a higher risk of pre-eclampsia, low birth weight, preterm birth, and other adverse obstetrical outcomes [14–17]. Furthermore, critical cases of pregnant women and newborn infants infected with COVID-19 might require intensive care unit admission, mechanical ventilation, or extracorporeal membrane oxygenation to manage severe respiratory failure conditions [18–20].

Despite remarkable advances in understanding the impact of COVID-19 on pregnancy and lactation, data remain sparse regarding the safe use of COVID-19 medications in this special population [21]. Currently, World Health Organization (WHO) has not approved any drug therapy interventions or established safe and effective for treating COVID-19 during pregnancy and lactation. In addition, many investigational drugs have been experimented with intermittently and as required for managing COVID-19 infection during pregnancy and lactation [21–25]. Therefore, healthcare professionals must know about the adverse outcomes of drugs used in pregnancy and lactation [26].

Several drug information resources are available in print and online, providing information on drug usage during pregnancy and lactation to enhance patient safety and achieve a better therapeutic outcome. However, easily accessible and reliable resources evaluating the safety of drugs used in pregnancy and lactation remain challenging [27]. The reaserch hypothesis that are the various drug information resources consistent in providing information related to various aspects of drug use in pregnancy and lactation?. To the best of our knowledge, even though studies evaluating the safety and efficacy profile of other therapeutic options for COVID-19 in pregnancy and lactation are available, investigations analyzing the consonance among the drug information resources are scanty [21–25]. This study aims to investigate the accuracy and consistency of information on COVID-19 medication use during pregnancy and breastfeeding. Additionally, we seek to evaluate the agreement between various drug information resources regarding these medications.

## Methods

This systematic comparative study was carried out using the various drug information resources widely used by healthcare professionals to concordance COVID-19 medications in pregnancy and lactation. Approval of the research proposal was obtained from the research and ethics committee of Ras Al Khaimah Medical and Health Sciences University (RAKMHSU-REC-108–2019-F-P). The study does not involve animal or human subjects and therefore does not require informed consent. We have considered printer resources such as drugs in pregnancy and lactation by Brigg's [28], Drugs for pregnant and lactating women by Carl P. Weiner [29], and Drugs during pregnancy and lactation – treatment options and risk assessment by Schaefer [30] for comparison. Additionally, subscription databases, namely Micromedex® [31], Portable Electronic Physician Information Database (PEPID^©^) [32], Up-to-date® [33], and readily available online tools such as Medscape.com [34] and Drugs.com [35] were also used. The online resource Drugs and Lactation Database (LactMed) [36], providing information on medications in lactation only, was also included.

Furthermore, information about COVID-19 repurposed medicines or agents that are investigated for the management of COVID-19 was collected from various guidelines and by carrying out a literature search in the databases PubMed, Google Scholar, Scopus, and Web of Science, which are updated till October 2021 [37–39]. We searched the electronic databases using a combination of Medical Subject Headings (MeSH) terms and keywords related to 'coronavirus disease and/or medications in pregnancy/lactation/breastfeeding' or 'COVID-19 and/or drugs in pregnancy/lactation/breastfeeding' or 'COVID disease and/or medications in pregnancy/lactation/ breastfeeding. In addition, references cited in these articles were also searched manually to identify further studies.

The research team carefully selected and listed COVID-19 medications used during pregnancy and lactation and was assessed by four expert reviewers. The reviewers consist of one obstetrician and gynecologist, two drug information experts, and a senior clinical pharmacist. The primary goal of the expert evaluation was to verify that all the selected drugs were pertinent and to pinpoint any supplementary drugs that needed to be added to the list. Three independent study investigators collected the medication list from each resource using a standard data collection form. Discussions resolved any disagreements between the investigators. Information on each medication was documented in an excel spreadsheet for further analysis.

Scope, completeness, and consistency were the three critical endpoints for evaluating the study objectives. The scope is defined as the "*Description of information related to pregnancy/lactation as an entry in the resource, calculated as a percentage of drug information that had an entry for each resource*." In comparison, completeness was defined as a resource containing clear and precise information discussing each of the identified elements, such as pregnancy (provides information on data related to animal/human studies along with clinical considerations and risk summary), lactation (contains data related to animal/human studies along with clinical considerations and risk summary during breastfeeding), Infertility (information related to the reproductive studies and fetal or infant risk), drug levels (information related to the quality or the amount of drug excreted or distributed into the breast milk) and pregnancy category (or) recommendation (data related to FDA pregnancy category or any other pregnancy recommendations). Finally, the completeness score was tallied as "a percentage of information with an entry describing every element individually" [40]. 

The total completeness score was computed by assigning one mark to each item. Furthermore, sum up the five items' scores to give a score between 0 and 5 for each information resource. For example, if any drug entry provides clear, precise details concerning all the five components, it will score five. On the other hand, if any drug provides information related to only pregnancy, lactation, and drug level and does not discuss the fertility and pregnancy category, it would acquire a score of three out of five.

To evaluate discrepancies related to the medicine use during pregnancy and lactation, the investigators collected the data for all the COVID-19 medications. They were then divided into six following categories, namely 'can be used,' 'individual benefit-risk assessment,' 'should not be used, 'trimester-specific information' (related to pregnancy), 'not classifiable,' 'no available information as derived by Frost Widnes and Jan Schott [41]. The research team also provided descriptions of each category for the proper classifications of the recommendations.

### Data analysis

Descriptive statistics, namely frequency and percentages were used to express scoring results for score, completeness, and discrepancies. The overall completeness was assessed using a median and interquartile range. Scope score was compared by using the McNemar test, and the Wilcoxon signed-rank test was used to analyze the overall completeness scores. A tier analysis was also carried out to group resources by related scope and completeness scores. Resources having the maximum score were compared with the following higher-ranking resources in a series until the difference in the score was statistically significant (*p* < 0.05).

When allocating categorical ratings, the Fleiss kappa (k) coefficient was used to examine the dependability of concordance between the investigators and the different drug information resources. The Fleiss kappa value's degree of agreement was calculated using Landis and Koch's criteria. A score of less than 0.0 indicates 'poor agreement,' 0.0—0.2 indicates slight agreement, 0.21–0.40 shows fair agreement, 0.41–0.60 indicates moderate agreement,0.61– 0.80 indicates substantial agreement, and scoring between 0.81–1.00 denotes almost perfect agreement [42]. Furthermore, a *p*-value of < 0.05 is computed for each kappa, demonstrating statistical significance.

We also compared the significance of discordant for pregnancy and lactation information among resources between medications that were newly introduced (lopinavir, remdesivir, immunoglobulin, favipiravir, casirivimab, imdevimab, ritonavir, ribavirin, anakinra, tocilizumab, and alteplase) and those that were already available (azithromycin, chloroquine, hydroxychloroquine, interferon, dexamethasone) by using the fisher's exact test.

Similarly, to investigate the discrepancies, information from the information resources was compared for the different categories, namely 'can be used,' 'individual benefit-risk assessment,' 'should not be used, 'trimester-specific information' (related to pregnancy), 'not classifiable' and 'no available information. This was also later analyzed by Fleiss's kappa (k) coefficient. All the collected data were analyzed using the Statistical Package for the Social Sciences (SPSS) version 27 software (IBM Corp., Armonk, NY).

## Results

Following review by the subject expert research team after referring to guidelines and scientific databases, 18 therapeutic options used for COVID-19 in pregnancy and lactation were confirmed for the analysis (Table 1). These were the most commonly used drugs for managing various symptoms associated with COVID-19 during the study period and were selected after reviewing the literature, guidelines, and databases. The list includes anti-viral medications, antibiotics, antimalarial agents, immunoglobulins, tissue plasminogen activators, monoclonal antibodies, steroids, and interferon, which have been used for various other indications. The selected drugs were assessed for various components of our study objectives. During the analysis, it was noted that recommendations were unavailable for the two medications from the selected resources, so the study was confined to 16 COVID-19 medicines used in pregnancy and lactation.

**Table 1:** List of medications recommended for the management of COVID-19 in pregnancy and lactation

Anakinra	Favipiravir	Nafamostat
Azithromycin	Hydroxychloroquine	Remdesivir
Alteplase	Immunoglobulin	Ribavirin
Casirivimab	Imdevimab	Ritonavir
Chloroquine	Interferon	Tocilizumab
Dexamethasone	Lopinavir	Umifenovir

### Scope scores

Among the subscription database, PEPID© and up-to-date had the highest scope score of 88.8 percent (16 out of 18), whereas Drugs.com was the top most among the free online (16 out of 18) and drugs used in pregnancy and lactation by Briggs and also by Schaefer placed high level among the textbooks resources (12 out of 18). However, these findings were not statistically significant compared to all the other resources (Table 2).

**Table 2:** Scope score for the drug information resources. (Scope scores were analyzed for the most commonly used drug information resources that provide information related to the safe use of drugs in pregnancy and lactation)

Drug Information resource	Type of drug information resource	Total number of drugs (*N* = 18)	
		**n**	**%**
PEPID	Database	16	88.88
Up-to-date	Database	16	88.88
Micromedex	Database	15	83.33
Drugs in Pregnancy & Lactation by Brigg's, 12^th^ Ed 2022, Wolters Kluwer Publication	Textbook	12	66.66
Drugs during Pregnancy & Lactation by Schaefer, 3^rd^ Ed 2015, Elsevier	Textbook	12	66.66
Drugs for Pregnancy & Lactating women by Weiner, 3^rd^ Ed 2019, Elsevier	Textbook	10	55.55
LactMed (https://www.ncbi.nlm.nih.gov/books/NBK547442/)	Online	16	88.88
Drugs.com (https://www.drugs.com/pregnancy/)	Online	16	88.88
Medscape.com (https://reference.medscape.com/)	Online	14	77.7

*Abbreviations: PEPID*© Portable Electronic Physician Information Database

### Completeness scores

The completeness score for pregnancy ranged from 87.5% (PEPID) to 100% (Micromedex, up-to-date, Medscape.com along with all the textbook resources), and for lactation, ranged from 75% (Drugs during pregnancy and lactation by Schaefer) to 100% (PEPID, Micromedex®, LactMed and Drugs for Pregnancy & Lactating women by Weiner). Information related to drug excretion into breast milk ranged from 25% to 62.5%, with drugs.com scoring highest among the drug information resources. Resources providing information about reproductive risk/fertility ranged from 56.2% (PEPID) to 100% (Drugs in Pregnancy & Lactation by Brigg's). However, considering the resources providing information related to pregnancy category/ recommendation, it ranged from 12.5% (PEPID) to 100% (Micromedex & Drugs in Pregnancy & Lactation by Brigg's), and few resources lack information related to pregnancy category/recommendation (Table 3).

**Table 3:** Completeness elements and overall completeness scores for the drugs with entries. (All the database resources were assessed for completeness, providing clear and precise information related to each identified element, such as pregnancy and lactation. Drug level, reproductive risk, and pregnancy category. The overall completeness was assessed by using the median and interquartile range. Data are presented as median (IQR) or n (%))

Resource	Number of drugs (n)	Pregnancy n (%)	Lactation n (%)	Drug level in breast milk n (%)	Reproductive risk/ Fertility n (%)	Pregnancy category/recommendation n (%)	Overall Completeness	
							**Median**	**IQR**
PEPID©	16	14 (87.5)	16 (100)	08 (50)	09 (56.2)	02 (12.5)	3	2—4
Micromedex®	15	15 (100)	15 (100)	04 (25.0)	11 (68.7)	15 (100)	4	3 – 5
Up to Date	16	16 (100)	15 (93.7)	05 (31.2)	13 (81.2)	00 (0)	3	3 – 4
Drugs in Pregnancy & Lactation by Brigg's	12	12 (100)	11 (91.6)	07 (58.3)	12 (100)	12 (100)	4	1—5
Drugs during Pregnancy & Lactation by Schaefer	12	12 (100)	9 (75.0)	07 (58.3)	09 (75.0)	00 (0)	3	0—4
Drugs for Pregnancy & Lactating women by Weiner	10	10 (100)	10 (100)	05 (50.0)	10 (100)	07 (70.0)	4	1—5
LactMed	16	00 (0)	16 (100)	07 (43.7)	00 (0)	00 (0)	1	1—2
Drugs.com	16	15 (93.7)	15 (93.7)	10 (62.5)	13 (81.25)	12 (75.0)	4	3—5
Medscape.com	14	14 (100)	11 (78.5)	04 (28.5)	09 (64.2)	00 (0)	2	2—4

*Abbreviations: PEPID*© Portable Electronic Physician Information Database

The overall completeness ratings for various drug information resources varied from 1 (IQR 1 to 2, LactMed) to 4 (IQR 3 to 5, Micromedex® and drugs.com). Resources were categorized into one to three tiers based on the scope scores and completeness. PEPID, UpToDate, and drugs.com are placed in the highest tier for the scope compared to other drug information resources. Therefore, the completeness tier analysis has to lead the way to three tiers, namely tier1 (Micromedex, drugs.com; *p* < 0.05 as compared to all remaining resources), tier 2 (Medscape.com, PEPID, Up-to-Date, Drugs During Pregnancy and Lactation by Schaefer; *p* < 0.05 as compared to all remaining resources) and tier 3 (LactoMed, Drugs in Pregnancy and Lactation by Briggs, Drugs for Pregnancy and Lactating women by Weiner) against all other resources for each comparison (Table 4).

**Table 4:** Tier analysis of the drug information resources based on scope and completeness. (The tiers analysis were carried out using the scope score and the completeness. Drug information resources with higher scope and completeness were placed in tier 1, followed by tier 2 and tier 3)

Tiers	Scope	Completeness
Tier 1	PEPID, Up-to-date, Drugs.com, LactoMed	Micromedex, Drugs.com
Tier 2	Micromedex, Medscape.com	Medscape.com, PEPID, Up-to-Date, Drugs during Pregnancy & Lactation by Schaefer
Tier 3	Drugs in Pregnancy & Lactation by Brigg's, Drugs during Pregnancy & Lactation by Schaefer, Drugs for Pregnancy & Lactating women by Weiner	LactoMed, Drugs in Pregnancy & Lactation by Brigg's, Drugs for Pregnancy & Lactating women by Weiner

### Inter-source reliability analysis

We observed 'slight' agreement (*k* value 0.0—0.2) among the drug information resources concerning the components' lactation' and 'pregnancy category/recommendation. However, for the information related to the component' pregnancy' and 'reproductive risk/fertility,' it was discovered that there is a 'fair' agreement between the various drug interaction sources. The strength of agreement for the element drug level in breast milk was moderate. The Fleiss kappa for overall completeness inter-rater agreement was shown to be a 'slight' agreement (Table 5).

**Table 5:** Inter reliability analysis for the concordance among the drug information resources studied. (The value of ‘k’ helps to assess the strength of agreement between the investigators and the different drug information resources)

Components of the drug information resources	Value of k^†^	95% Confidence Interval	*P*-value *	Strength of Agreement
Pregnancy	0.238	0.235 – 0.241	0.0001	Fair
Lactation	0.108	0.106 – 0.111	0.009	Slight
Drug level in breastmilk	0.445	0.442 – 0.447	0.0001	Moderate
Reproductive risk / Fertility	0.291	0.288 – 0.294	0.0001	Fair
Pregnancy category/recommendation	0.141	0.136 – 0.146	0.075	Slight
Overall Completeness	0.047	0.045 – 0.048	0.001	Slight

^*^p value < 0.05 is statistically significant

^†^*k* < 0.2 signifies poor agreement

We analyzed the data from multiple medication information resources to investigate discrepancies in the recommendations for the components between the newer and the older drugs from the selected COVID-19 medications in our study using fisher's exact test. Except for the online resource drugs.com (*p* > 0.05), there was a statistically significant difference in the different components across the various drug information resources (*p* < 0.05) between older and newer drugs (Table 6).

**Table 6:** Discrepancy analysis of recommendations among drug information resources for newer and old medications used for COVID-19 management in pregnancy and lactation. (The discordance among the various drug information resources was studied for the various newer and older COVID-19 medications used in pregnancy and lactation)

		**PEPID (*n* = 16)**	**Micromedex (*n* = 16)**	**Up-to-date (*n* = 15)**	**Drugs for Pregnancy & Lactating women (Weiner) (*n* = 10)**	**Drugs in Pregnancy & Lactation (Schaefer) (*n* = 12)**
Newer drugs v/s Older drugs	Fishers exact	6.88	11.14	10.34	6.32	13.26
	Sig. (2-tailed)	**0.020**^**b**^	**0.002**^**a**^	**0.004**^**a**^	**0.038**^**b**^	** < 0.001**^**a**^
		**Drugs in Pregnancy & Lactation (Brigg's) (*n* = 12)**	**LactoMed (*n* = 16)**	**Drugs.com (*n* = 16)**	**Medscape.com** **(*n* = 14)**	
Newer drugs v/s Older drugs	Fishers exact	7.82	9.35	4.03	11.17	
	Sig. (2-tailed)	**0.018**^**b**^	**0.005**^**a**^	**0.333**	**0.003**^**a**^	

^a^ statistically significant at 0.01 level (2-tailed)

^b^ statistically significant at 0.05 level (2-tailed)

Similarly, we have conducted an inter-reliability analysis to assess the observer agreement to determine the reliability among the various raters for the recommendation categories of the different COVID-19 medications used in our study. We have identified a 'moderate' strength of agreement for the recommendation categories 'can be used,' 'should not be used, and 'no available information. Furthermore, for the categories' benefit-risk assessment' and 'not classifiable,' we observed a 'fair' and 'poor; strength of agreements, respectively (Table 7).

**Table 7:** Inter reliability analysis for the observer agreement among the recommendation categories for the various COVID-19 medications studied. (The various recommendation categories were assessed for the strength of agreement between the investigators and the different drug information resources)

Recommendation categories	Value of k†	95% Confidence Interval	*P*-value *	Strength of Agreement
Can be used	0.422	0.420 – 0.425	< 0.001	Moderate
Individual benefit – risk assessment	0.215	0.213 – 0.218	< 0.001	Fair
Should not be used	0.415	0.413 – 0.417	< 0.001	Moderate
Not classifiable	-0.039	-0.042 – -0.037	0.319	Poor
No available information	0.554	0.551 – 0.556	< 0.001	Moderate

^*^p value < 0.05 is statistically significant

^†^*k* < 0.2 signifies poor agreement

**Fig. 1:**
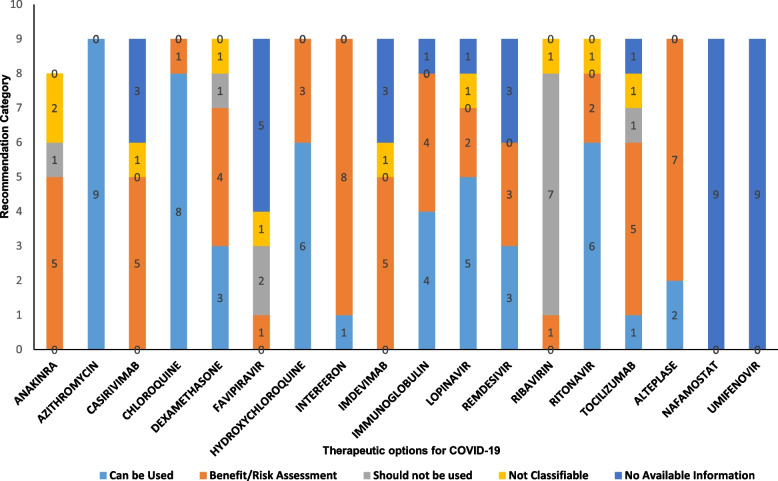
Distribution of the recommendation categories in drug information resources for the COVID-19 medications in pregnancy and lactation. (All the COVID -19 medications that were included in the study were plotted graphically for the different recommendation categories to assess the consistency among the drug information resources)

The recommendations from the various drug information resources for the selected COVID-19 medications in pregnancy and lactation were classified according to the different recommendation categories to assess the consistency among the resources. The category 'trimester-specific information' was excluded from the discrepancy analysis as no data could be classified into this section considering the information available from the different drug information resources. The study identified homogeneity ranging from 66.6%—100% for fewer drugs between the data resources regarding the recommendations for some of the categories from the selected COVID-19 medications. This includes azithromycin (100%), chloroquine (88.8%), hydroxychloroquine, and ritonavir (66.6%), where the majority of raters and the data resources agreed to recommend the category 'can be used' in COVID-19 during pregnancy and lactation (Fig. 1).

## Discussion

Prescribing medications during pregnancy and lactation is always more complex for practicing healthcare professionals to prevent maternal and fetal health problems. Therefore, drug information resources play a vital role in helping physicians while prescribing medications, especially the healthcare practitioner dealing with childbirth taking into account the safety of the mother and the fetus [43]. So, it is essential to consult the available references for information related to its use in COVID-19 during pregnancy and lactation. Therefore, the present study systematically compared the therapeutic options for COVID-19 concerning its use in pregnancy and lactation by referring to the various drug information resources.

The present study evaluated the different information resources for the scope, completeness, and inconsistency of COVID-19 medications in pregnancy and lactation. According to the study's findings, the scope for accessing information was minimal, with only five out of nine resources presenting more than 80% of selected COVID-19 medications. Among the various drug information resources reviewed, PEPID, Up-to-date, and drugs.com had the highest information in scope score. These findings were consistent with the study assessing the uniformity of information across different drug information databases related to drug-drug interactions carried out by Shariff et al. This observation demonstrated that PEPID and UpTo Date had the most drug pair entries with excellent scope scores [44]. Similarly, a study assessing the various tools for screening drug interaction of oral oncolytic drugs reported that Lexi comp and Drugs.com were the highest-ranking subscriptions and free online resources compared to other drug interaction tools [45].

Also, Choi Hee et al. found that Lexi-comp had the most significant quantum of information out of the five resources when they looked at how similar the recommendations for dose adjustments in renal failure were across different drug information resources [46]. Compared to our research, this variation might be attributed to varied study aims and information resources. Our research noted that no drug information resources could provide collective information for all the selected COVID-19 medications. In addition, among all the various information resources, only 9 out of the total 18 medications were similarly identified in resources. The subscription-based databases and the free online tools provided information for more than 80% of the drugs included in our study compared to text references, ranging from 55%-66%. We have also identified that the newer medications like remdesivir, favipiravir, and monoclonal antibody drugs had no entry in any of the included text references but were accessible online and in subscription databases.

The present study also showed that Micromedex, Drugs.com, and the text references related to pregnancy and lactation by Brigg's et al. and Weiner et al. scored higher in overall completeness than PEPID and Up-to-date. The text reference for pregnancy and lactation by Schaefer et al. analyzing the different components of the overall completeness showed that subscription databases such as PEPID, Micromedex, and Up-to-date and the text references related to pregnancy and lactation by Briggs et al. along with the online tools namely drugs.com could be considered as a resource of choice to scrutinizing the information about the safety of the drugs in pregnancy and lactation. Additionally, the drug and lactation database (LactMed), a free online tool, can be included as a reference for exploring the information related to lactation. It was also noted that the information related to the drug excretion or distribution into the breast milk found in the references was low, ranging from 25—62.5% among all the resources.

Most of the resources highlighted as available evidence are inconclusive or insufficient for determining the amount, quantity, or level of drug that enters breast milk and determining the fetal risk while breastfeeding. Similarly, the information related to the reproductive risk/fertility was not evident from the available resources with varying information reporting as no evidence of impaired fertility/fetal risk from the animal studies or risk for major fetal malformations was non-significant or data is insufficient/ not available/ inconclusive/unknown to assess the potential effects on fertility from exposure. However, our study suggests preferring text references (75% -100%) over the subscription and online tools (68% -81%) for the effect of the drugs on reproductive risk/fertility. In addition, the study noticed a disparity among the drug information resources regarding pregnancy category/recommendation.

Micromedex defines the pregnancy category as contraindicated, fetal harm has been demonstrated, may cause fetal harm, fetal risk cannot be ruled out, and fetal risk is minimal. In contrast, the text reference by Briggs et al. defines pregnancy recommendations as compatible, probably compatible, suggest risk, suggest high risk, suggest medium risk, suggest low risk, have no relevant animal data, and are contraindicated.

Moreover, tools such as PEPID, Drugs.com, and the text reference by Schaefer et al. follow the FDA pregnancy category (A, B, C, D, and X), sometimes ambiguous and hazy. Drug information resources like UpToDate, Medscape and the text reference by Schaefer have not specified any pregnancy category in their drug monograph. This heterogeneity creates uncertainty among obstetricians in clinical decision-making about whether to use it during pregnancy and who needs it when there is no other choice.

This calls for a policy effort to standardize certain elements and principles before initiating drug therapy to ensure that the treatment is safe, rational, and scientifically sound. Providing encyclopedic information to the clinicians from the resources helps the physicians to select safer and better quality medicines from the available treatment options. Much is needed for obstetrics, and pediatric drug therapy as precise and consistent information helps practicing healthcare professionals avoid medications that are likely to cause harmful effects on the mother and the fetus. The inter-rater reliability analysis using the Fleiss kappa analyzes the agreement between the raters, and we have used it to evaluate the consensus among the resources. The study found an overall 'slight' strength of agreement among the information resources for the various components of pregnancy and lactation assessed by the Fleiss kappa (K) score. For example, the Fleiss kappa coefficient for the category 'lactation' and 'pregnancy category/recommendation' < 0.2, indicating slight agreement.

Conversely, the kappa coefficient for the category 'pregnancy' and 'reproductive risk/fertility was found to be in the range of 0.21–0.4, which demonstrates a fair degree of agreement, and for the category' drug level in breast milk,' The strength of agreement among the various information resources reviewed was determined to be moderate. A study carried out by Cheng et al*.* evaluating consistencies between drug information resources and the manufacturer's prescribing information (PI) identified varying degrees of discordances with the boxed warning in the prescribing information in comparison with all other resources (*p* < 0.0001). In addition, the overall interrater Fleiss kappa agreement was excellent (kappa = 0.86) [47].

Studies have shown inconsistencies among healthcare professionals' resources providing drug information [48, 49]. To explore the inconsistency in detail, we have conducted the discrepancy analysis for various components among the drug information resources by comparing the newer and older drugs from the selected COVID-19 medications using fisher's exact test. We have observed a statistically significant difference (*p* < 0.05) between the newer and the older drugs for the different components provided by all the drug information resources except drugs.com (*p* > 0.05). Information related to the older drugs in most of the resources, including the text references, provides in-depth details on various components such as pregnancy safety, clinical data related to lactation, the effect of the drug distribution into breast milk, reproductive potential/infertility risk and the pregnancy category/recommendations. However, the information was provided in the subscription and free online tools for the newer drugs compared to the text references. Nevertheless, most information provided was superficial and incomplete, with insufficient data and inconclusive evidence. This knowledge gap about safety and efficacy creates uncertainty in the prescribers' clinical decision-making for pregnancy and lactation.

The observer agreement for the various recommendation categories of COVID-19 medication was examined using the inter-rater reliability analysis by measuring Fleiss kappa (k). The 'k' co-efficient for the recommendation categories 'can be used,' 'should not be used,' and 'no available information was between 0.41–0.60, indicating moderate agreement. This observation suggests that most raters agree with recommendation categories according to the report specified in the drug information resources. For example, azithromycin, all the drug information resources agreed with sufficient data that this drug could be used safely in pregnancy and lactation. Likewise, ribavirin was the majority of the drug information resources, and the raters admit its use is contraindicated during pregnancy.

Similarly, the kappa coefficient for the category' individual risk–benefit assessment' was 0.215, which shows a 'fair' strength of agreement. For the type 'not classifiable, it was less than zero suggesting a 'poor' strength of agreement for the different drug resources studied. This disparity in the strength of agreement is mainly for the newer drugs that the raters suggest for individual risk–benefit assessment, mainly due to a lack of available data to strengthen the evidence about its safety in pregnancy. Correspondingly insufficient or little information from the drug information resources insists the raters categorize as 'not classifiable.' A study by Norby et al. recognized that inconsistency between the online information resources regarding drug information is common in pregnancy and lactation and suggests consonance in information to avoid contradictory messages [50]. Another study by Frost Widnes and Jan Schjott also reported a considerable difference between the resources providing information about their use in pregnancy. The k coefficient for the observer agreement in their study was found to be 0.67, suggestive of a 'good' strength of agreement [41].

Our study's strengths include previous studies evaluating the recommendations from the available drug information resources lacking locally and globally to the best of the author's knowledge, especially in pregnancy and lactation. Second, use the most relevant and highly considered printed resources, subscription-based and freely available online tools of nine different databases to evaluate information among the drug information resources for scope, completeness, and discrepancy.

Our study has several strengths that set it apart from previous research. Firstly, to our knowledge, no prior studies have evaluated drug information resources for COVID-19 medications in pregnancy and lactation. Secondly, we thoroughly assessed information from relevant printed resources, subscription-based, and freely available online tools from nine databases. This allowed us to compare and assess the scope, completeness, and discrepancies among various drug information resources. However, our study has some limitations. Drug information resources update their information at different intervals, which was not accounted for in our research, and some selected medication information from free online tools was ambiguous and imprecise, leading us to categorize it as 'not classifiable.' Lastly, information provided in some resources was disorganized, making it difficult for healthcare professionals to use efficiently.

## Conclusion

Our study observed disagreement among the resources in recommendations on using COVID-19 treatment options in pregnancy and lactation. The study also reports discrepancies in the information related to pregnancy, lactation, drug level, reproductive risk, and pregnancy recommendations among the resources directing to refer to more than one resource for information about the safe and quality use of medications in this special population. The presentation emphasizes the need for standardized, comprehensive, accurate, and evidence-based information related to pharmacotherapy which is of paramount importance to prescribers in making informed choices in pregnancy and lactation. Prompt access to high-quality, unbiased, and updated recommendations in clinical practice may not only help optimize the health outcome of the mother and the fetus but also minimize the exposure to the harmful effects of medications. The data presented in this study will serve as a basis for carrying out similar studies assessing the consistency among the drug information resources for the drugs used in managing chronic conditions and illnesses that may appear during pregnancy and lactation.

### Use of artificial intelligence in writing the manuscript [51]

 Artificial intelligence was not used.

## Supplementary Information

**Additional file 1.**

**Additional file 2.**

**Additional file 3.**

**Additional file 4.**

## Data Availability

All the authors declare that data and materials supporting the results reported in the manuscript are available from the corresponding author at reasonable request.
